# RNAi-mediated downregulation of *AcCENH3* can induce in vivo haploids in onion (*Allium cepa* L.)

**DOI:** 10.1038/s41598-024-64432-7

**Published:** 2024-06-24

**Authors:** Tushar K. Manape, Viswanathan Satheesh, Saravanakumar Somasundaram, Parakkattu S. Soumia, Yogesh P. Khade, Pawan Mainkar, Vijay Mahajan, Major Singh, Sivalingam Anandhan

**Affiliations:** 1 ICAR-Directorate of Onion and Garlic Research, Rajgurunagar, Pune, Maharashtra 410505 India; 2 ICAR-National Institute of Plant Biotechnology, Pusa Campus, New Delhi, 110012 India; 3 Present Address: Genome Informatics Facility, Office of Biotechnology, Iowa State University, Ames, IA 50010 USA; 4 Present Address: Leibniz Institute of Plant Genetics and Crop Plant Research (IPK) Gatersleben, 06466 Seeland, Germany

**Keywords:** Genome elimination, Centromere specific histone3, Segregation distortion and haploid induction, Biotechnology, Molecular biology, Plant sciences

## Abstract

Haploid induction (HI) holds great promise in expediting the breeding process in onion, a biennial cross-pollinated crop. We used the CENH3-based genome elimination technique in producing a HI line in onion. Here, we downregulated *AcCENH3* using the RNAi approach without complementation in five independent lines. Out of five events, only three could produce seeds upon selfing. The progenies showed poor seed set and segregation distortion, and we were unable to recover homozygous knockdown lines. The knockdown lines showed a decrease in accumulation of *AcCENH3* transcript and protein in leaf tissue. The decrease in protein content in transgenic plants was correlated with poor seed set. When the heterozygous knockdown lines were crossed with wild-type plants, progenies showed HI by genome elimination of the parental chromosomes from *AcCENH3* knockdown lines. The HI efficiency observed was between 0 and 4.63% in the three events, and it was the highest (4.63%) when E1 line was crossed with wildtype. Given the importance of doubled haploids in breeding programmes, the findings from our study are poised to significantly impact onion breeding.

## Introduction

Haploid induction (HI) plays a pivotal role in the field of plant genetics and breeding. Doubled haploids significantly reduce the time to achieve high levels of homozygosity, making the development of true-breeding lines quick and efficient^1^. Traditional methods of creating homozygous lines require several generations of self-pollination, but with haploids, this can be achieved in a single generation through HI and chromosome doubling. These haploids bolster plant breeding by enabling the consistent creation of genetically superior varieties and hybrids^2^.

HI can be achieved through the in vitro regeneration of gametes^3^. Further, there have been reports of in vivo methods, including wide hybridization^4^ and utilization of HI lines within species like maize^5^. In vivo induction methods, which are reported in specific crops, often lack broad applicability and may not be suitable for a wide range of crop species. The breakthrough invention of CENH3-mediated genome elimination offers great promise for the induction of haploids in different plant species^6^. The CENH3 protein is a histone H3 variant that localizes at the centromere and interacts directly with the components of the kinetochore complex during cell division. It is highly divergent and rapidly evolving across species as its N-terminal tail is highly variable in length and sequence^7^. The classic approach involved complementing the CENH3 knockout mutant with GFP-tailswap-CENH3^6^. Several modifications to the original methods were implemented in plants such as *Arabidopsis*, maize, and wheat^8–13^. These studies reported the use of non-synonymous mutations on the histone fold domain (HFD), sequence modification in the N-terminal tail of CENH3, or replacement of CENH3 from other related species. In addition, heterozygous knockout null mutant was also used to induce haploids^12,14^.

Onion is a biennial crop that completes its life cycle in a year or two. It is a cross-pollinated crop with a high level of inbreeding depression, which renders the development of genetically uniform (inbred) onion lines challenging. Inducing haploids in onion is quicker and more cost-effective compared to conventional breeding methods. Although the production of haploid and doubled haploid onions via in vitro gynogenesis is available^15^, its adoption in breeding programmes is limited due to genotypic variability, cost and access to resources for breeders. In this context, availability of HI lines will not only assist in the development of haploids but will also facilitate trait transfers such as male sterility, gene pyramiding^1^ and one-step genome editing^16^. In this study, we show that unlike *Arabidopsis*^17^ and maize^18^, a nominal reduction in *CENH3* expression using RNAi results in genome elimination when crossed with wildtype (WT) onion. The method described here has potentially wide application as this can be applied to crops where CRISPR/*Cas9*-based knockout generation is not feasible.

## Results and discussion

### Generation and molecular confirmation of *AcCENH3* knockdown lines

**Figure 1:**
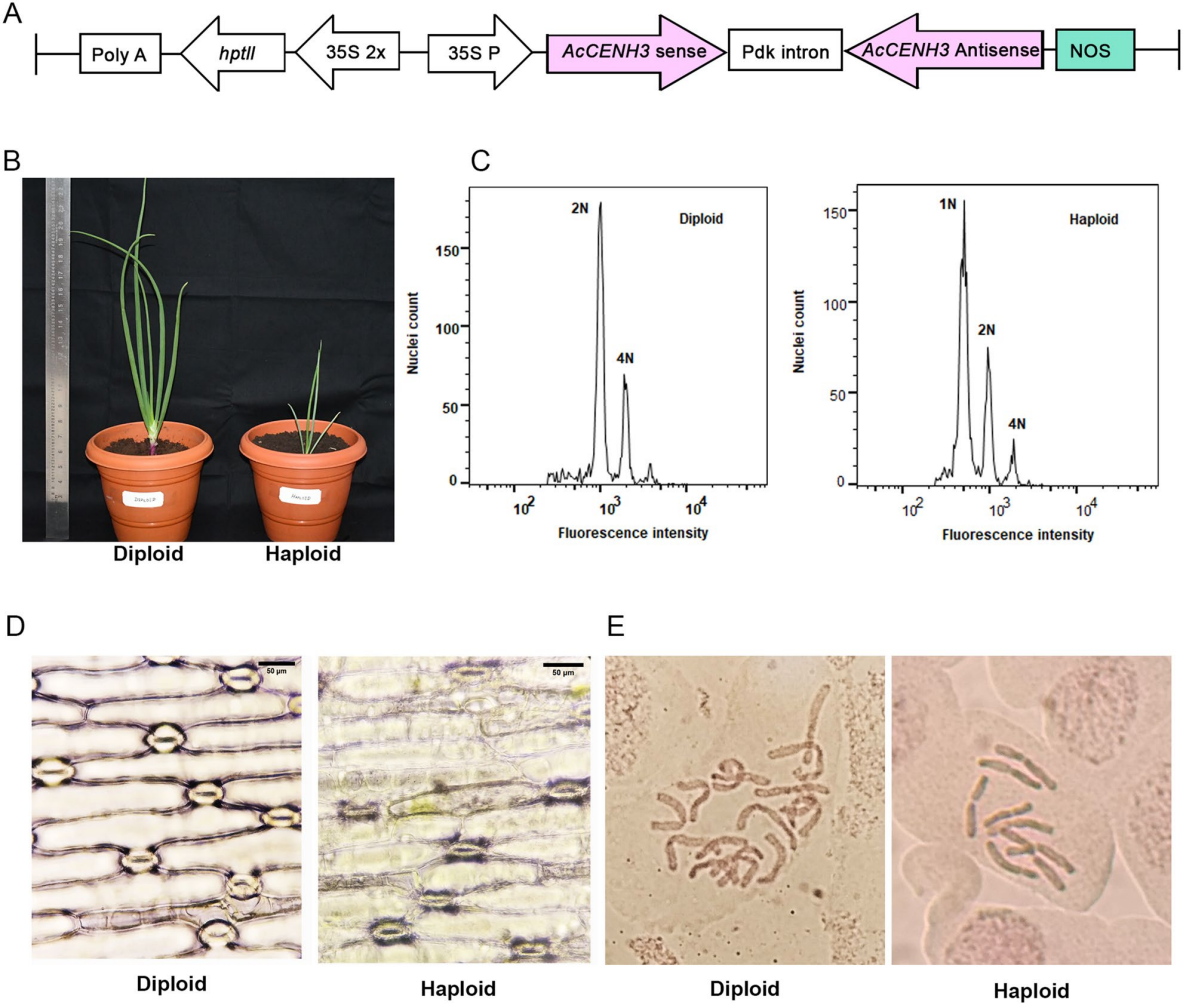
Construct used and confirmation of haploidy in progenies. (**A**) Schematic representation of *AcCENH3*-RNAi construct showing the CaMV35S (35S) promoter-driven sense and anti-sense fragments interposed with a Pdk intron. (**B**) Haploids plants have a shorter stature. (**C**) Flow cytometric analysis of haploids showing diploid plants with peaks at 2N and 4N. Haploid plants have 1N and 2N peaks. (**D**) Haploid plants show a reduction in stomatal size (Scale bar, 50 µm). (**E**) Chromosome spreads from the root tips of onion reveal 16 chromosomes in diploids and 8 in haploids.

A hairpin RNAi construct targeting *AcCENH3* was designed using a 397 bp sense/antisense fragment of *AcCENH3* and designated as *AcCENH3*-RNAi (Fig. 1A, Supplementary Table S1). A total of 3942 embryogenic calli of onion cultivar Bhima Super was transformed with *AcCENH3*-RNAi construct by using the *Agrobacterium*-mediated transformation protocol. Five independent *AcCENH3* knockdown lines regenerated, with a transformation efficiency of 0.13%, which was lower than the 1% observed in our previous study using a GUS construct^19^. Putative transgenic lines were validated by PCR amplification with T-DNA-specific primers for *hpt*II, sense and antisense strands of *AcCENH3*-RNAi construct (Supplementary Table S1 and Supplementary Fig. 1). Additional thermal asymmetric interlaced PCR (TAIL-PCR) analyses of T_0_ plants confirmed single copy insertion of transgene and identified the T-DNA-plant genome junctions (Supplementary Fig. 2). Further analysis of the junction sites revealed that none of these insertions disrupted functional genes, confirming the independence of all 5 events (designated as E1–E5). Based on these insertion sites, event-specific markers were designed (Supplementary Table S1). These markers were also used to screen for the zygosity of plants in the T_1_ generation. All five independent events were acclimatized under greenhouse conditions, where they successfully formed bulbs. These bulbs were then planted in the subsequent season to complete their biennial life cycle. Unfortunately, all plants from E4 failed to survive. Among the surviving events, E3 did not produce seeds (Supplementary Fig. 3). Thus, for subsequent analyses, T_1_ plants from E1, E2, and E5, along with bulb-derived T_0_ plants from E3, were used.

### Biased segregation distortion in *AcCENH3* knockdown lines

When the selfed progenies of events E1, E2, and E5 were screened using T-DNA specific primers, the population was found to be not in confirmation with the segregation ratio of 3:1 (Supplementary Table S2). We observed 24.24%, 23.53%, and 37.5% positive plants out of totals of 99, 85, and 24 plants in events E1, E2, and E5, respectively, which is a significant deviation from the expected frequency of 75% (Supplementary Table S2). Further, PCR analysis using event-specific T-DNA and plant junction markers in the T_1_ population of *AcCENH3* knockdown lines E1, E2 and E5, revealed the presence of heterozygous and azygous plants. We observed a single homozygous plant in E2, which died early in its development (Supplementary Fig. 4A,B and Supplementary Table S3). This indicated that the survival of homozygous knockdown lines was severely affected probably due to higher suppression of *AcCENH3* than in heterozygous lines. F_1_ generation obtained from the reciprocal crosses of *AcCENH3* knockdown lines (E1 and E2) with WT did not conform to the 1:1 segregation of the transgene. The E5 test cross (with WT) population conformed to 1:1 segregation but segregation distortion was observed in the selfed population. Interestingly, the transgene segregation distortion is more pronounced when the two events, E1 and E2, were used as the male parent. In maize, the inheritance of the knockout allele from the female parent (25%) was higher than that in the male parent (12.1%)^12^. We also observed that *AcCENH3*-RNAi transgene inheritance was higher from the female parents than males. In the events E1 and E2, the inheritance of the transgene was much higher from the female parent with a 5% lower inheritance observed from the male parent (Supplementary Table S4). CENH3 is essential for male gamete maturation^20^, and reduction or loss of CENH3 can significantly impact transgene inheritance from the male gamete^14,21,22^. In summary, our findings demonstrate a significant deviation from the expected segregation ratios only in lines E1 and E2. As we were not able to recover homozygous knockdown lines, we used heterozygous *AcCENH3* knockdown lines in all further analyses.

### *AcCENH3-*RNAi transgenic lines show decreased *AcCENH3* transcript and protein levels

To assess the impact of the dsRNA hairpin RNAi cassette on native *AcCENH3* transcripts and protein levels, we analysed the heterozygous knockdown lines using quantitative RT-PCR, and ELISA (T_0_ plants were used for E3). qRT-PCR showed that the transcript levels of native *AcCENH3* were significantly reduced in all knockdown lines compared to the WT. The relative downregulation of *AcCENH3* transcripts in the RNAi transgenic events E1, E2, E3, and E5 was 0.74, 0.79, 0.59 and 0.89-fold, respectively, compared with WT (Table 1 and Supplementary Fig. 5A). ELISA was carried out with protein extracted from leaf tissue to quantify the AcCENH3 protein levels from knockdown lines E1, E2, E3 and E5. ELISA results showed that native AcCENH3 protein levels in all transgenic events decreased compared to the WT. The relative protein levels were 77.84%, 81.50%, 71.80% and 87.83% of WT AcCENH3 in knockdown lines of E1, E2,E3 and E5, respectively (Table 1; Supplementary Fig. 5B). Our findings from qRT-PCR, and ELISA analyses consistently indicated that decrease in *AcCENH3* transcript levels are commensurate with reduction in AcCENH3 protein levels. A substantial reduction of *CENH3* transcript was achieved in *A. thaliana* (27–43%)^21^, cotton (over 80%)^23^ and maize (21–65%)^18^. It is interesting to note that plants with *CENH3* knockout alleles in heterozygous state in *A. thaliana* and maize also survive^6,12,14^. Thus, it indicates these species i.e., Maize and *A. thaliana* can tolerate substantial reduction in CENH3 transcript. Segregation distortion and nonrecovery of homozygous lines for *AcCENH3-*RNAi locus in onion shows that slight reduction in CENH3 could affect its survival. This could be possibly due to insufficiency of AcCENH3 chromatin to support normal segregation of large chromosomes in onion knockdown lines.

**Table 1:** Quantitative parameters in *AcCENH3*-RNAi knockdown onion lines.

Event	*AcCENH3* transcript abundance by qRT-PCR	AcCENH3 protein level by ELISA	Seed set efficiency in selfing of T_1_	Seed set efficiency in outcrossing RNAi as female parent	Seed set efficiency in crossing RNAi as male parent
Bhima Super (WT)	100 ± 6.9	96.51 ± 0.90	99.62 ± 4.14	98.65 ± 5.02	96.73 ± 4.21
E1	73.67 ± 2.3	77.84 ± 1.13	27.10 ± 4.28	49.32 ± 3.79	37.89 ± 5.05
E2	78.67 ± 2.7	81.50 ± 0.98	29.86 ± 5.01	67.34 ± 2.72	61.82 ± 5.28
E3	58.5 ± 1.5	71.80 ± 1.82	–	–	–
E5	89.33 ± 2.0	87.83 ± 0.71	66.5 ± 2.97	81.57 ± 5.63	83.76 ± 4.37

### *AcCENH3* knockdown lines show poor seed set in self-fertilized and outcross progeny

In the second season, all T_1_ plants from all events produced seeds upon self-fertilization except for E3 (Table 1 and Supplementary Figs. 3, 6). The T_2_ seed yield per umbel from the flowering transgenic events was lower than that from the WT. The seed set efficiency in transgenic events E1, E2, and E5 was 27.10%, 29.86%, and 66.50%, respectively, relative to the WT (Table 1 and Supplementary Fig. 6). Furthermore, a correlation analysis between AcCENH3 quantity (OD values from ELISA) and relative seed set efficiency revealed a correlation coefficient of 0.962. This suggested that a decrease in the AcCENH3 protein impacted the seed set (Supplementary Fig. 7). The seed set efficiency upon outcrossing was significantly lower in the transgenic lines with WT compared to the WT × WT crosses. When *AcCENH3* knockdown lines served as the female parent, relative seed set efficiency was 49.32%, 67.34%, and 81.57% in events E1, E2, and E5, respectively (Table 1 and Supplementary Fig. 8). Conversely, when *AcCENH3* knockdown lines were used as the male parent, the relative seed set efficiency was 37.89%, 61.82%, and 83.76% for events E1, E2, and E5, respectively (Table 1 and Supplementary Fig. 8).These findings further suggested that the knockdown of *AcCENH3* affected the seed set. The differential seed set could be one of the reasons for the biased segregation distortion of the transgene observed in the reciprocal crosses. Our observations are similar to the previous studies, emphasizing that a decline in CENH3 levels is associated with a decrease in seed set efficiency both during selfing^21^ and when crossed with WT^6,14,18^.

### *AcCENH3* knockdown lines show in vivo haploid induction

In our study, the F_1_ population derived from reciprocal crosses of *AcCENH3* knockdown lines (heterozygous) with WT was analysed using FCM for estimating the ploidy level (Fig. 1C). Progenies from events E1 and E2 showed haploid induction (Table 2). In these events, the HI efficiency (HIE) ranged from 2.04 to 4.63%. The highest HIE was observed in E1 (4.63%). While aneuploids were observed in progenies of all events, there was no HI in the progenies of E5 and WT × WT crosses. Haploids identified by FCM were further confirmed for the ploidy by cytological staining of root tips (Fig. 1E). The haploids showed poor growth when compared to diploid progenies (Fig. 1B). Microscopic analysis of leaf surface revealed a 41.25% decrease in the size of stomata in haploid plants, indicating cell size reduction (Fig. 1D and Supplementary Fig. 9). Our result was in accordance with Foschi and co-workers^24^. Interestingly, the HIE was noticeably higher when the *AcCENH3* knockdown parent served as the male, but statistical analysis analysis of male *vs* female based HI using Fisher’s exact test showed no significant difference (Table 2). A comparative analysis of HIE across various plant species, revealed that *A. thaliana* recorded the highest HIE at approximately 40%^6^. In crop plants, HIE was comparatively lower than *A. thaliana*. Cotton exhibited the highest HIE (8%)^23^, followed by wheat (7%)^13^ and maize (5.2%)^12^. In other species, rice demonstrated an HIE of 1%, similar to cucumber and melon, while tomato had a slightly higher rate of 2.3%, and these rates pertain to EMS mutants^25^. In this study, we achieved an HIE of 4.63% in onion, which is close to the efficiencies observed in maize.

**Table 2:** HI efficiency of *AcCENH3*-RNAi knockdown onion lines.

Cross (♀) × (♂)	No. of plants tested	No. of haploids	No of aneuploids	HIE (%)	*p*-value^#^	*p*-value^$^
E1 × WT	112	3	2	2.68	0.014*	0.7216
E2 × WT	98	2	2	2.04	0.047*	1.0000
E5 × WT	52	0	1	0	1	–
WT × E1	108	5	3	4.63	0.0007*	–
WT × E2	90	2	1	2.22	0.041*	–
WT × E5	59	0	3	0	1	–
WT × WT	350	0	0	0	–	–

^#^*p*-value for pairwise comparison of crosses involving events with WT and WTxWT; ^$^*p*-value for pairwise comparison of crosses involving events as female or male with WT; *indicates significance at *p* ≤ 0.05.

Since the breakthrough study of Ravi and Chan, several methods have been devised to develop CENH3-based HI lines^6^. Among them, a simple approach has been to maintain the knockout allele under a heterozygous state for inducing haploids upon crossing with WT lines^12^. The heterozygous knockout allele for *CENH3* in maize might have led to a reduction in the centromere size by dilution of CENH3 in the cellular environment and could have resulted in genome elimination in a competitive environment when crossed with WT^12,26^. Similar observations were reported in *A. thaliana i.e.,* heterozygous knockout when crossed with WT resulted in 0.83% of haploids in the progeny^14^. These results indicate that *Arabidopsis* can tolerate a significant reduction in CENH3. On the other hand, in onion, even a slight reduction in CENH3 levels leads to seed sterility and HI (Tables 1 and 2). These observations in *Arabidopsis*, maize and onion suggest the existence of a species-specific critical threshold for CENH3 chromatin. Our approach to develop knockdown-based HI lines builds upon the work by Wang and co-workers in maize^12^, offering an alternative when the generation of knockouts is not feasible due to a higher CENH3 threshold.

In this report, we demonstrate successful generation of HI lines in onions. The induction rates are comparable to those achieved through in vitro gynogenesis^27^. However, our in vivo HI method offers breeders a cost-effective approach to produce haploids from both male and female gametes. This method also eliminates the dependency on genotype-specific regeneration processes. Furthermore, our approach facilitates the transfer of traits such as male sterility and genome editing across different genotypes in a single generation, bypassing the labour-intensive backcross breeding. As a result, this methodology holds significant promise for transforming onion breeding practices.

## Methods

### Plasmid construction and onion transformation

A 397 bp sense and antisense strand of *AcCENH3* (Accession No. OQ281757) was PCR-amplified from the first strand of cDNA of onion cv. B. Super (Supplementary Table S1). It was then cloned into an intermediate vector, pHANNIBAL^28^, at the *Xho*I-*Kpn*I and *Hind*III-*Xba*I sites, respectively. The *AcCENH3*-RNAi cassette was extracted and subsequently cloned into the destination vector, pCAMBIA1305.1 (GenBank accession no. AF354045), at the *Nco*I and *Pml*I restriction sites, positioned under the CaMV35s promoter and Tnos terminator sequences and designated as *AcCENH3*-RNAi construct. Eight-week-old embryogenic calli, induced from the radicle of the onion cv. B. Super seeds, were transformed using the *AcCENH3*-RNAi plasmid, following the *Agrobacterium*-mediated transformation protocol^19^. Multiple plantlets that emerged from a single callus were considered as the same event and were acclimatized under greenhouse conditions (23 ± 2 °C with a 16 h light/8 h dark cycle). These acclimatized events were labelled as—E1, E2, E3, E4, and E5. Bulbs were harvested upon reaching maturity, replanted in the subsequent season, and T_1_ seeds were collected. Seed set upon selfing was recorded as the number of seeds per umbel in both T_1_ and WT plants. T_1_ flowers were crossed with the WT parent reciprocally, and the number of seeds obtained for the total number of flowers used in crossing was also documented. Voucher specimens of the onion seedling was preserved as herbarium in the germplasm repository of the Center with voucher specimen number: DOGR Voucher 01 following standard procedures.

### PCR analysis

T_0_, T_1_, and F_1_ plants were analysed by PCR to verify the presence of T-DNA (including sense strand, antisense strand, and *hpt*II gene) in their genomic DNA (see Supplementary Table S1). The specific primers and PCR conditions utilized for these analyses are detailed in the Supplemental Table S1. The integration of the *AcCENH3*-RNAi T-DNA into the onion genome was ascertained via TAIL–PCR^29^. This was subsequently used to discern whether T_1_ plants were homozygous or hemizygous. For qRT-PCR analysis, total RNA was extracted from the young leaves of 45-day-old seedlings. Total RNA was extracted from three seedlings of all independent events (except event #3, where 2 plants were used) and wild-type plants using a RNA extraction kit (Macherey–Nagel, cat# 740120.50). The cDNA was then synthesized using the SuperScript IV Reverse Transcriptase kit (Invitrogen, cat# K1622), following the manufacturer’s guidelines. The qRT-PCR procedure was carried out on a QIAquant 96 5plex real-time system (Qiagen), employing a TB Green RT-PCR kit (Takara, cat# RR820a). Relative gene expression levels were determined based on the 2^−ΔΔCt^ method, using onion ubiquitin as the internal control (Supplementary Table S1).

### *AcCENH3* protein detection

Total protein was extracted from young leaves of 45-day-old seedlings using the native buffer from Pierce Plant Total Protein Extraction Kit (ThermoScientific, cat# A44056), and quantified by Pierce Rapid Gold BCA Protein Assay Kit (ThermoScientific, cat# A53226). Three seedlings each from all independent events (except event #3, where 2 plants were used) and WT plants were used for protein extraction. An indirect assay was performed in 96-well plates (Genaxy cat# GEN-MTP-96F-S) coated with 100 µl of 1 mg/ml total protein (as an antigen) diluted in coating buffer (50 mM carbonate-bicarbonate buffer, pH 9.4), and incubated overnight at 4 °C in dark condition. Plate wells were washed once with 200 µl of PBS-T (phosphate-buffered saline with 0.2% Tween 20), blocked with 200 µl of 5% non-fat dry skimmed milk in PBS-T and incubated overnight at 4 °C in dark condition to reduce non-specific background signals. Plate wells were washed twice with PBS-T and incubated overnight with a 100 µl diluted (0.045 µ/mL) AcCENH3-specific antibody. Plate wells were washed three times with PBS-T, and diluted (0.1 µg/mL) 100 µl of AP-conjugated and goat anti-rabbit IgG (H + L) secondary antibody (Invitrogen USA, cat # T2191) were added, incubated for 2 h at room temperature and later washed four times with PBS-T. 100 µl of pNPP chromogenic substrate was added to the wells and incubated for 30 min before the reaction was stopped by adding 50 µL 2N NaOH and read using a SPECTROstar Nano microplate reader at an optical density of 405 nm.

### Chromosome staining

Root tips (1–2 mm) from in vitro-grown, two-week-old seedlings were fixed in acetic alcohol (ethanol: glacial acetic acid at a 3:1 ratio) for 3 h at 4 °C. These were then treated with preheated 1N HCl for 2 min and stained with aceto-carmine stain (10 g/L) for another 2 min. After staining, the root tips were placed on a glass slide and examined under a Leica compound microscope (LEICA DM 2500).

### Stomatal measurement

The foliar epidermis of eight-week-old F_1_ plants was removed using the ‘peeling’ technique^30^. Each print (approximately 1 × 1 cm in size) was observed using a Leica DM 2500 compound microscope at 400× magnification. The size (width and length) of the stomata was measured on these prints using Leica Image Manager Software V. 5.0.R247.

### Flow cytometry

Approximately 25–30 mg of leaf tissue from four-week-old seedlings was finely chopped with a razor blade in 1 ml of ice-cold Galbraith buffer. The resulting nuclei suspension was filtered through a 40 µm nylon mesh filter, treated with RNase A, and stained with 10 µl of propidium iodide (50 µg/ml) for 10 min in darkness while incubating on ice. The isolated nuclei suspension was then analyzed using flow cytometry at a low flow rate (15 µl/min) on the PE-A channel (585/42 nm). For each sample, a total of 20,000 events were recorded using a BD FACSCanto II instrument. The acquired data were analyzed with FlowJo v.10.9 software.

### Statistical analysis

Transformation efficiency was expressed as a percentage, determined by counting the number of transgenic events generated from the total number of co-cultivated calli^19^. ELISA, and qRT-PCR were performed in triplicate. Transgene segregation in T_1_ and F_1_ plants was assessed using the chi-square (χ^2^) test. Outcrossing fertility efficiency was expressed as a percentage based on the number of seeds set out of the total number of flowers crossed. All data are presented as mean ± SE and were subjected to the Shapiro–Wilk test for normality assessment and analysis of variance (ANOVA) using the SAS Base v9.2 (SAS Institute, NC, USA). Statistical significance was determined using Tukey’s test at *p* ≤ 0.05. Haploid induction efficiency in F_1_ progenies was determined as a percentage, based on the number of haploids identified from the total plants analyzed. Statistical significance was estimated using Fisher’s Exact Test by pair-wise comparison of HI in crosses involving event with WT *vs* WTxWT and also events as female *vs* male using GraphPad online calculator (https://www.graphpad.com/quickcalcs/contingency1/).

### Ethical approval

This article does not contain any studies with human participants or animals performed by any of the authors. The plant collection and use was in accordance with all the relevant guidelines.

### Supplementary Information

Supplementary Information.

## Data Availability

The datasets generated during the current study are available in the NCBI GenBank repository with accession number OQ281757; OR581163; OR581164; OR581165; OR581166 and OR581167 (Supplementary Table S5).
