# Supplementary material for: RNAi-mediated downregulation of AcCENH3 can induce in vivo haploids in onion (Allium cepa L.)
Source: Sci Rep. 2024 Jun 24;14:14481. doi: 10.1038/s41598-024-64432-7 (PMC11196721; doi:10.1038/s41598-024-64432-7)
Supplement: Supplementary file 1 — Supplementary Information. [file 41598_2024_64432_MOESM1_ESM.docx]

**Supplementary Information**

**RNAi-mediated downregulation of *AcCENH3* can induce *in vivo* haploids in onion (*Allium cepa* L.)**

Tushar K. Manape^1^, Viswanathan Satheesh^2#^, Saravanakumar Somasundaram^1##^, Parakkattu S. Soumia^1^, Yogesh P. Khade^1^, Pawan Mainkar^1^, Vijay Mahajan^1^, Major Singh^1^ & Sivalingam Anandhan^1*^

1. ICAR-Directorate of Onion and Garlic Research, Rajgurunagar, Pune, 410505, Maharashtra, India
2. ICAR-National Institute of Plant Biotechnology, Pusa Campus, New Delhi 110012, India

^#^ Present address: Genome Informatics Facility, Office of Biotechnology, Iowa State University, Ames, Iowa 50010, USA

^##^ Present address: Leibniz Institute of Plant Genetics and Crop Plant Research (IPK) Gatersleben, 06466, Seeland, Germany

*Corresponding author: anandhans@gmail.com, anandhan.s@icar.gov.in


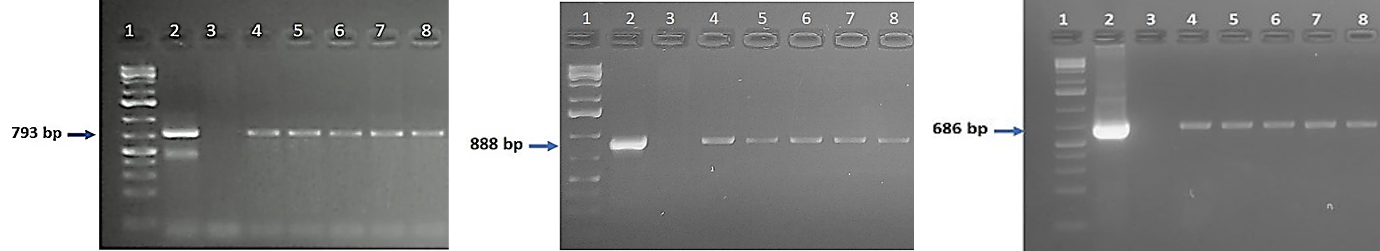


**C**

**B**

**A**

**Supplementary Fig. 1 PCR analysis of putative *AcCENH3*-RNAi transgenic plants (T_0_) to confirm the presence of A. sense strand (793bp), B. Antisense strand (888bp), C. *hptII* gene (686bp) sequences**

(Lane 1: 1 kb ladder, 2: *AcCENH3*-RNAi plasmid, 3: Bhima Super (WT), 4 to 8: 5 independent transgenic events). The expected amplicon size (bp) is marked as a blue arrow. To amplify sense and antisense strand of *AcCENH3*, PDK intron from silencing construct was included, and to avoid amplification from native *AcCENH3*.


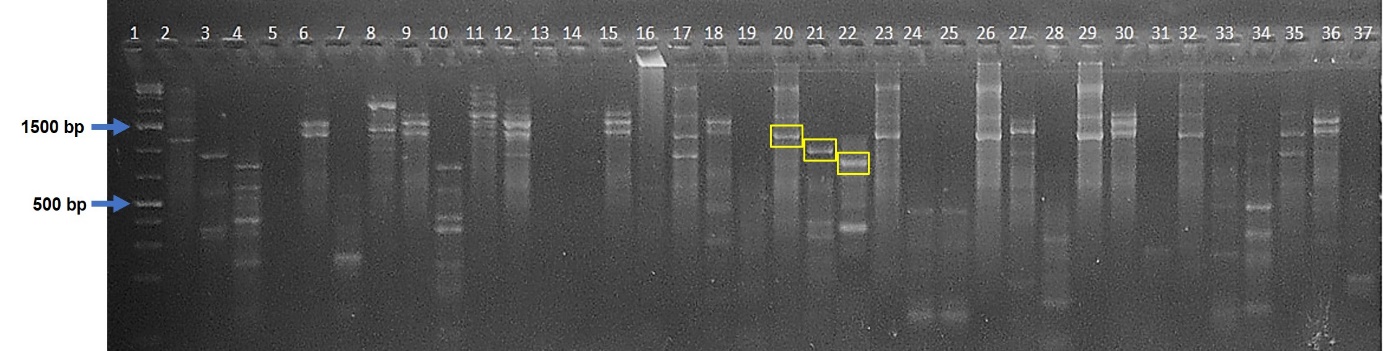


**Supplementary Fig. 2** **Representative image of TAIL PCR of T_0_ *AcCENH3*-RNAi knockdown lines.**

TAIL-PCR carried out with three sets of nested primers located on left border (LB) or right border (RB) of T-DNA sequence and arbitrary primers; Lane 1- 1 kb plus ladder; Lane 2, 5, 8, 11, 14, 17- shows primary PCR product with left border specific (LBS) and 6 different degenerate primers; 3, 6, 9, 12, 15, 18- shows secondary PCR product with LBS and 6 different degenerate primers; 4, 7, 10, 13, 16, 19- shows tertiary PCR product with LBS and 6 different degenerate primers; 20, 23, 26, 29, 32, 35- shows primary PCR product with right border specific (RBS) and 6 different degenerate primers; 21, 24, 27, 30, 33, 36- shows secondary PCR product with RBS and 6 different degenerate primers; 22, 25, 28, 31, 34, 37- shows tertiary PCR product with RBS and 6 different degenerate primers. Yellow marked PCR products had shown expected size difference-based distance between the three nested primers located on RB or LB of T-DNA sequence.

**Supplementary Fig. 3** **Information on stage-wise *AcCENH3*-RNAi knockdown lines.**

Onion is a biennial crop. The crop undergoes bulbing in the first season and bulbs are replanted to induce flowering and seed set in the next season. Transgenic plantlets from tissue culture were hardened in pots to produce bulbs and bulbs are harvested and replanted to produce seeds. The schematic figure represents fate of transgenic events developed in the study. X-indicates crop progress in the designated event was stopped in earlier stage and not included in the current experiment.


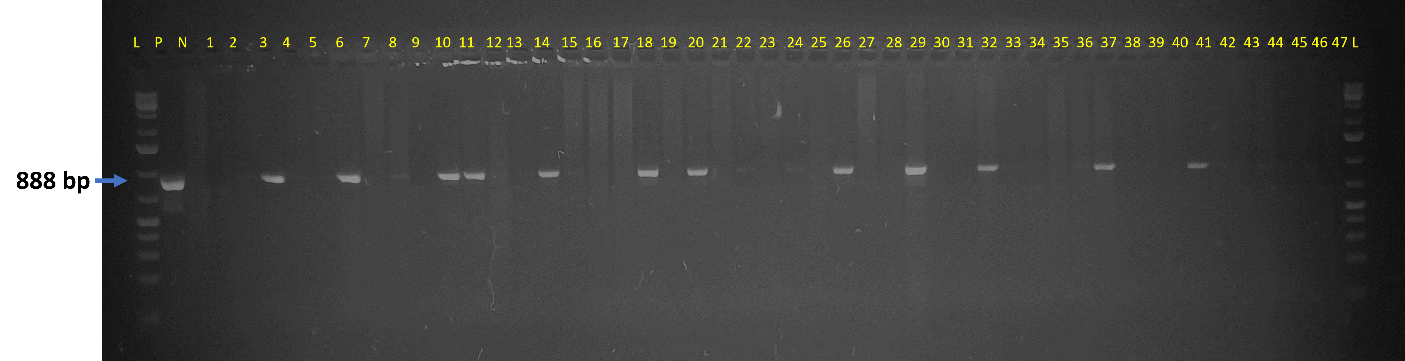


**Supplementary Fig. 4A Representative image of segregation analysis of *AcCENH3*-RNAi transgene in *AcCENH3*-RNAi knockdown lines using transgene-specific primer set.**

Antisense strand-specific primer set was used to confirm the presence of *AcCENH3*-RNAi cassette in F_1_ plants of *AcCENH3*-RNAi knockdown lines.

[Lane 1: 1 kb plus ladder, P: *AcCENH3*-RNAi plasmid, N: Bhima Super DNA (WT), 1 to 47: F_1_ plants of *AcCENH3*-RNAi event #1]. The amplicon of size 888bp shown with blue arrow.


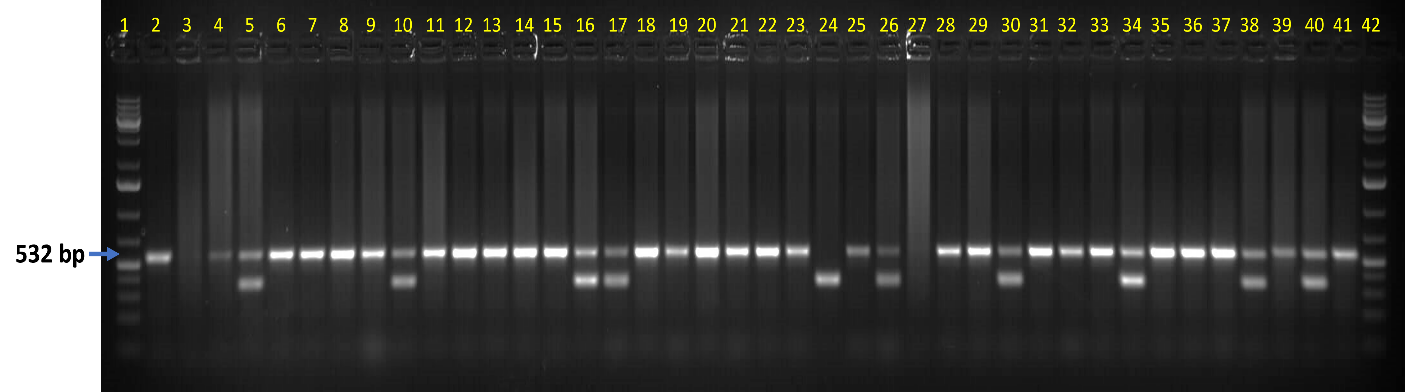


**Supplementary Fig. 4B Representative image of segregation analysis of *AcCENH3*-RNAi transgene in *AcCENH3*-RNAi knockdown lines using event specific primer set.**

A PCR was performed using T-DNA specific primers and site of insertion specific primer to identify homozygous and heterozygous lines of *AcCENH3*-RNAi knockdown lines.

[Lane 1 & 42: 1 kb ladder, 2 & 41: Bhima Super (WT), 3 to 40: T_1_ plants of RNAi event #2]. A single amplicon of size 532 bp (lane 2) and 371 bp (lane 24) indicate azygous and homozygous, respectively, while two amplicons of size 532 and 371 bp represent heterozygous for transgene locus.

**Supplementary Fig. 5A Relative *AcCENH3* transcript abundance in the *AcCENH3*-RNAi knockdown transgenics.**

Total RNA was extracted from leaves of Bhima Super (WT) and T_1_ plants (T_0_ plant of event #3) expressing *AcCENH3*-RNAi construct. qRT-PCR was carried out to estimate relative abundance of *AcCENH3* and *AcUbiquitin* was used as an internal control. The relative transcript abundance was assessed using 2^-ΔΔCt^ method. The relative abundance was expressed as fold change over control (WT). The onion cultivar Bhima Super was used as control. Data is represented as mean ± SE. The data was analyzed using completely randomized design (CRD, n=14, p=0.000).

**Supplementary Fig. 5B Relative AcCENH3 protein abundance in the *AcCENH3*-RNAi knockdown lines.**

Total protein was isolated from leaves of WT and T_1_ transgenic lines (T_0_ plant of event #3) and equal quantity of protein (100 µg) from each sample was used for ELISA using rabbit-AcCENH3 specific antibody. Relative expression was calculated as percentage over WT. Data is represented as mean ± SE. The data was analyzed using CRD (n=42, p=0.000).

**Supplementary Fig. 6 Seed set efficiency upon selfing of the T_1_ population of *AcCENH3*-RNAi knockdown lines.**

The umbels of T_1_ plants of event #1, #2, #5 and Bhima Super (WT) were covered with bags and allowed to self. The number of seeds per umbel were recorded in triplicate and expressed as relative seed set over control (WT). Data is represented as mean ± SE. The data was analyzed using CRD (n=16, p=0.000).

**Supplementary Fig. 7 Linear regression analysis of *AcCENH3*-RNAi transgenic lines.**

A regression analysis was carried out between the relative seed set efficiency of selfed WT and T_1_ plants (T_0_ in case of E#3) and the levels of AcCENH3 protein (OD values @ 405nm in ELISA). The linear regression analysis revealed a strong direct relationship between AcCENH3 protein level and relative seed set efficiency, with a calculated R^2^ value of 0.925 and correlation R value of 0.962 (n=14, p=0.000).

**Supplementary Fig. 8 Seed set efficiency upon outcrossing of the T_1_ population of *AcCENH3*-RNAi transgenic lines.**

Reciprocal crosses were made with T_1_ *AcCENH3*-RNAi knockdown lines and wild-type and relative seed set efficiency was calculated as percentage over the number of seeds obtained from control crosses (WT X WT). Data is represented as mean ± SE. The data was analyzed using CBD (n=24; p=0.000).

**Supplementary Fig. 9 Comparative analysis of stomatal aperture size in diploid and haploid plants.**

Stomatal size was measured in length and width in diploid (Bhima Super) and haploid Plants. Haploid plants showed reduction in size**.** Data is represented as mean ± SE. The data was analyzed using CRD (n=12, p=0.000).

**Supplementary Table S1 List of primers used in the study**

| **Primer Name** | **Primer sequences (5'-3')** | **Amplicon size and region** | **PCR conditions** |
| --- | --- | --- | --- |
| **Primers used for cloning transgenic confirmation** | |  |  |
| AcCenh3 F | GACGAAGCTGGTCCTTCAAC | 397 bp of *AcCenH3* sense strand sequence | 1 cycle of 94 °C for 5 min; 35 cycles of 94 °C for 45 sec, 55 °C for 45 sec, 72 °C for 30 sec, and 1 cycle of 72 °C for 10 min |
| AcCenh3 R | TAGCACCACCAATCCTCCTC |  |  |
| Sense F | CCGCTCGAGCCATGGGACGAAGCTGGTCCTTCAAC | 397 bp of *AcCenH3* sense strand sequence | 1 cycle of 94 °C for 5 min; 35 cycles of 94 °C for 45 sec, 55 °C for 45 sec, 72 °C for 30 sec, and 1 cycle of 72 °C for 10 min |
| Sense R | CGGGGTACCTAGCACCACCAATCCTCCTC |  |  |
| Antisense F | CCCAAGCTTTAGCACCACCAATCCTCCTC | 397 bp of *AcCenH3* antisense strand sequence | 1 cycle of 94 °C for 5 min; 35 cycles of 94 °C for 45 sec, 57 °C for 45 sec, 72 °C for 45 sec, and 1 cycle of 72 °C for 10 min |
| Antisense R | CTAGTCTAGACACGTGGACGAAGCTGGTCCTTCAAC |  |  |
| CaMV35s F | CCAACCACGTCTTCAAAGCAAG | 793 bp (partial sequence of CaMV35s prom + sense strand+ partial sequence of *pdk* intron | 1 cycle of 94 °C for 3 min; 35 cycles of 94 °C for 45 sec, 58 °C for 45 sec, 72 °C for 60 sec and 1 cycle of 72 °C for 10 min |
| pDK intron R | TCTTCTTCGTCTTACACATCACTTG |  |  |
| pDK intron F | TGCTAATATAACAAAGCGCAAGAT | 888 bp (partial sequence of *pdk* intron + antisense strand+ partial sequence of Tnos terminator | 1 cycle of 94 °C for 3 min; 35 cycles of 94 °C for 45 sec, 58 °C for 45 sec, 72 °C for 60 sec and 1 cycle of 72 °C for 10 min |
| tNOS R | TCCTAGTTTGCGCGCTATATTTTG |  |  |
| Hpt F | GACGTCTGTCGAGAAGTTTC | 686 bp of internal sequence of *hptII* | 1 cycle of 94 °C for 3 min; 35 cycles of 94 °C for 45 sec, 57 °C for 45 sec, 72 °C for 45 sec and 1 cycle of 72 °C for 10 min |
| Hpt R | GCCTCCAGAAGAAGATGTTG |  |  |
| **Primers used for qRT-PCR** |  |  |  |
| qUBI F | TCTGATTGTGCTTGTGGTGGTT | 131 bp of *AcUbiquitin* | 1 cycle of 95 °C for 2 min; 40 cycles of 95 °C for 10 sec, 58 °C for 10 sec  Melt curve 60 °C to 95 °C. |
| qUBI R | AAAGCGGAAATTAAGATGATGTTGC |  |  |
| qAcCENH3 F | CAGTTTTCCCAACGGCAGAA | 250 bp of N-tail region *AcCENH3* |  |
| qAcCENH3 R | TCGAGCTCCAGTCCCTCTAT |  |  |
| **Primers used for detection of homozygous/heterozygous transgenic lines** | | | |
| **Primer Name** | **5’ to 3’ sequence** | **Amplicon size and region** | **PCR conditions** |
| **RNAi Event 1** | | | |
| RB F3 | GCATGACGTTATTTATGAGATGG | In hetero: 703 & 652 bp  In azygous: 703 bp | 1 cycle of 95 °C for 5 min; 35 cycles of 94 °C for 45 sec, 58 °C for 45 sec, 72 °C for 45 sec and 1 cycle of 72 °C for 10 min |
| Insert E1 R | GCATCCATAGCGACTATCGCTA |  |  |
| Insert E1 F | GGAAAATCTAACTTGGATTTGG |  |  |
| **RNAi Event 2** |  |  |  |
| RB F3 | GCATGACGTTATTTATGAGATGG | In hetero: 572 & 355 bp  In azygous: 572 bp | 1 cycle of 95 °C for 5 min, 35 cycles of 94 °C for 45 sec, 56 °C for 45 sec,72 °C for 1 min and 1 cycle of 72 °C for 10 min |
| Insert E2 R | GCATTTGGAGATTTCGTCGAAG |  |  |
| Insert E2 F | CTTGCATCCCTGGTATGGTATC |  |  |
| **RNAi Event 3** |  |  |  |
| LB F2 | CAAAATCCAGTACTAAAATCCAGATCC | In hetero: 316 & 338 bp  In azygous: 338 bp | 1 cycle of 95 °C for 5 min, 35 cycles of 94 °C for 45 sec, 60 °C for 45 sec, 72 °C for 45 sec and 1 cycle of 72 °C for 10 min |
| Insert E3 R | GCAAAGCAAGATGAGCACGAG |  |  |
| Insert E3 F | CTACTAGTGCTTAGCTCTATGG |  |  |
| **RNAi Event 4** |  |  |  |
| RB R1 | GGTGTCATCTATGTTACTAGATCG | In hetero: 418 & 546 bp  In azygous: 540 bp | 1 cycle of 95 °C for 5 min, 35 cycles of 94 °C for 45 sec, 52 °C for 45 sec, 72 °C for 45 sec and 1 cycle of 72 °C for 10 min |
| Insert E4 R | CACTTGGTTTATGTAACGATCCCGA |  |  |
| Insert E4 F | GAAATTATGTTAAAGTCCCTAGTCTTG |  |  |
| **RNAi Event 5** |  |  |  |
| LB F1 | GTGTTATTAAGTTGTCTAAGCGTC | In hetero: 324 & 287 bp  In azygous: 324 bp | 1 cycle of 95 °C for 5 min, 35 cycles of 94 °C for 45 sec, 60 °C for 45 sec, 72 °C for 45 sec and 1 cycle of 72 °C for 10 min |
| Insert E5 R | GATTTAAAGGGTAATCGTTTATCTC |  |  |
| Insert E5 F | TAATTATCTTTACGCAATTAATTACCTTG |  |  |
| **Primers for cloning and transgenics confirmation** | | | |
| **Primer Name** | **Primer sequences (5'-3')** | **Amplicon size and region** | **PCR conditions** |
| Sense F | CCGCTCGAGCCATGGGACGAAGCTGGTCCTTCAAC | 397 bp of *AcCenH3* sense strand sequence | 1 cycle of 94 °C for 5 min; 35 cycles of 94 °C for 45 sec, 55 °C for 45 sec, 72 °C for 30 sec, and 1 cycle of 72 °C for 10 min |
| Sense R | CGGGGTACCTAGCACCACCAATCCTCCTC |  |  |
| Antisense F | CCCAAGCTTTAGCACCACCAATCCTCCTC | 397 bp of *AcCenH3* antisense strand sequence | 1 cycle of 94 °C for 5 min; 35 cycles of 94 °C for 45 sec, 57 °C for 45 sec, 72 °C for 45 sec, and 1 cycle of 72 °C for 10 min |
| Antisense R | CTAGTCTAGACACGTGGACGAAGCTGGTCCTTCAAC |  |  |
| CaMV35s F | CCAACCACGTCTTCAAAGCAAG | 793 bp (partial sequence of CaMV35s prom + sense strand+ partial sequence of *pdk* intron | 1 cycle of 94 °C for 3 min; 35 cycles of 94 °C for 45 sec, 58 °C for 45 sec, 72 °C for 60 sec and 1 cycle of 72 °C for 10 min |
| pDK intron R | TCTTCTTCGTCTTACACATCACTTG |  |  |
| pDK intron F | TGCTAATATAACAAAGCGCAAGAT | 888 bp (partial sequence of *pdk* intron + antisense strand+ partial sequence of Tnos terminator | 1 cycle of 94 °C for 3 min; 35 cycles of 94 °C for 45 sec, 58 °C for 45 sec, 72 °C for 60 sec and 1 cycle of 72 °C for 10 min |
| tNOS R | TCCTAGTTTGCGCGCTATATTTTG |  |  |
| Hpt F | GACGTCTGTCGAGAAGTTTC | 686 bp of internal sequence of *hptII* | 1 cycle of 94 °C for 3 min; 35 cycles of 94 °C for 45 sec, 57 °C for 45 sec, 72 °C for 45 sec and 1 cycle of 72 °C for 10 min |
| Hpt R | GCCTCCAGAAGAAGATGTTG |  |  |
| **Primers used for qRT-PCR** |  |  |  |
| qUBI F | TCTGATTGTGCTTGTGGTGGTT | 131 bp of *AcUbiquitin* | 1 cycle of 95 °C for 2 min; 40 cycles of 95 °C for 10 sec, 58 °C for 10 sec  Melt curve 60 °C to 95 °C. |
| qUBI R | AAAGCGGAAATTAAGATGATGTTGC |  |  |
| qAcCENH3 F | GTGGTCCTTCAACACCAGTAAC | 184 bp of N-tail region *AcCENH3* |  |
| qAcCENH3 R | GGAGCAGCAGGAATCAGTAATTC |  |  |
| **Primers for detection of homozygous/heterozygous transgenic lines** | | | |
| **Primer Name** | **5’ to 3’ sequence** | **Amplicon size and region** | **PCR conditions** |
| **Event 1** |  |  |  |
| RB F3 | GCATGACGTTATTTATGAGATGG | In hetero: 703 & 652 bp  In azygous: 703 bp | 1 cycle of 95 °C for 5 min; 35 cycles of 94 °C for 45 sec, 58 °C for 45 sec, 72 °C for 45 sec and 1 cycle of 72 °C for 10 min |
| Insert E1 R | GCATCCATAGCGACTATCGCTA |  |  |
| Insert E1 F | GGAAAATCTAACTTGGATTTGG |  |  |
| **Event 2** |  |  |  |
| RB F3 | GCATGACGTTATTTATGAGATGG | In hetero: 572 & 355 bp  In azygous: 572 bp | 1 cycle of 95 °C for 5 min, 35 cycles of 94 °C for 45 sec, 56 °C for 45 sec,72 °C for 1 min and 1 cycle of 72 °C for 10 min |
| Insert E2 R | GCATTTGGAGATTTCGTCGAAG |  |  |
| Insert E2 F | CTTGCATCCCTGGTATGGTATC |  |  |
| **Event 3** |  |  |  |
| LB F2 | CAAAATCCAGTACTAAAATCCAGATCC | In hetero: 316 & 338 bp  In azygous: 338 bp | 1 cycle of 95 °C for 5 min, 35 cycles of 94 °C for 45 sec, 60 °C for 45 sec, 72 °C for 45 sec and 1 cycle of 72 °C for 10 min |
| Insert E3 R | GCAAAGCAAGATGAGCACGAG |  |  |
| Insert E3 F | CTACTAGTGCTTAGCTCTATGG |  |  |
| **Event 4** |  |  |  |
| RB R1 | GGTGTCATCTATGTTACTAGATCG | In hetero: 418 & 546 bp  In azygous: 540 bp | 1 cycle of 95 °C for 5 min, 35 cycles of 94 °C for 45 sec, 52 °C for 45 sec, 72 °C for 45 sec and 1 cycle of 72 °C for 10 min |
| Insert E4 R | CACTTGGTTTATGTAACGATCCCGA |  |  |
| Insert E4 F | GAAATTATGTTAAAGTCCCTAGTCTTG |  |  |
| **Event 5** |  |  |  |
| LB F1 | GTGTTATTAAGTTGTCTAAGCGTC | In hetero: 324 & 287 bp  In azygous: 324 bp | 1 cycle of 95 °C for 5 min, 35 cycles of 94 °C for 45 sec, 60 °C for 45 sec, 72 °C for 45 sec and 1 cycle of 72 °C for 10 min |
| Insert E5 R | GATTTAAAGGGTAATCGTTTATCTC |  |  |
| Insert E5 F | TAATTATCTTTACGCAATTAATTACCTTG |  |  |

**Supplementary Table S2 Segregation analysis of *AcCENH3-*RNAi transgene in T_1_ *AcCENH3*-RNAi transgenic lines.**

| **Transgenic line** | **Total plants analysed** | **Antisense strand positive** | **Antisense strand negative** | **χ ^2^** | **Fitness to 3:1** | **p** |
| --- | --- | --- | --- | --- | --- | --- |
| E1 | 99 | 24 | 75 | 136.03 | No | 0 |
| E2 | 85 | 20 | 65 | 120.098 | No | 0 |
| E5 | 24 | 9 | 15 | 18.00 | No | 0.00002209 |

Segregation analysis of T_1_ generation of 3 *AcCENH3*-RNAi knockdown lines. The segregation analysis with test of goodness of fit to ratio 3:1 indicates segregation distortion in transgenic events #1, #2 and #3. χ^2^_0.05/df1_=3.814

**Supplementary Table S3 Segregation analysis for zygosity of transgene locus of *AcCENH3*-RNAi in T_1_ transgenic lines.**

| **Transgenic line** | **Total plants analysed** | **Homozygous** | **Heterozygous** | **Azygous** | **χ^2^** | **Fitness to 1:2:1** | **^#^p** |
| --- | --- | --- | --- | --- | --- | --- | --- |
| E1 | 99 | 0 | 24 | 75 | - | No | 3.76 |
| E2 | 85 | 1 | 19 | 65 | 122.365 | No | 0 |
| E5 | 24 | 0 | 9 | 15 | - | No | 0.005 |

Segregation analysis for independent assortment of T_1_ generation of 3 *AcCENH3*-RNAi knockdown lines. The segregation analysis with test of goodness of fit to ratio 1:2:1 indicates segregation distortion in transgenic lines. χ^2^_0.05/df2_=5.991. **^#^** Fisher’s Exact Test was performed for E1 and E5, because the number of homozygous lines obtained was <1.

**Supplementary Table S4 Segregation analysis of *AcCENH3*-RNAi transgene in F_1_ *AcCENH3*-RNAi knockdown lines.**

| **Transgenic line** | **Total plants analysed** | **Antisense strand positive** | **Antisense strand negative** | **χ^2^** | **Fitness to 1:1** | **p** |
| --- | --- | --- | --- | --- | --- | --- |
| E1 as **♀** | 40 | 12  (30.0%) | 28 | 6.400 | No | 0.0114 |
| E1 as **♂** | 80 | 20  (25.05) | 60 | 20.000 | No | <0.0001 |
| E2 as **♀** | 31 | 10  (32.25%) | 21 | 3.903 | No | 0.0482 |
| E2 as **♂** | 95 | 26  (27.26%) | 69 | 19.463 | No | <0.0001 |
| E5 as **♀** | 22 | 9  (40.91%) | 13 | 0.727 | Yes | 0.3938 |
| E5 as **♂** | 29 | 11  (37.93%) | 18 | 1.690 | Yes | 0.1936 |

Segregation analysis of F_1_ generation of the transgenic lines. The segregation analysis with fitness to ratio 1:1 indicates segregation distortion in transgenic events #1 and #2. χ^2^_0.05/df1_=3.814. Numbers in parenthesis indicate the percentage of transgenic-positive plants.

**Supplementary Table S5 Genbank accession numbers provided by the NCBI.**

| **Events** | **Genbank accession numbers** |
| --- | --- |
| E1 | OR581163 |
| E2 | OR581164 |
| E3 | OR581165 |
| E4 | OR581166 |
| E5 | OR581167 |
